# In vivo rescue of genetic dilated cardiomyopathy by systemic delivery of nexilin

**DOI:** 10.1186/s13059-024-03283-x

**Published:** 2024-05-23

**Authors:** Yanjiao Shao, Canzhao Liu, Hsin-Kai Liao, Ran Zhang, Baolei Yuan, Hanyan Yang, Ronghui Li, Siting Zhu, Xi Fang, Concepcion Rodriguez Esteban, Ju Chen, Juan Carlos Izpisua Belmonte

**Affiliations:** 1 Gene Expression Laboratory, Salk Institute for Biological Studies, La Jolla, CA 92037 USA; 2 Altos Labs, San Diego, CA 92121 USA; 3 Department of Cardiology, Translational Medicine Research Center, Laboratory of Heart Center, Zhujiang Hospital, Southern Medical University, Guangzhou, 510280 China; 4 Guangdong Provincial Biomedical Engineering Technology Research Center for Cardiovascular Disease, Guangzhou, 510280 China; 5 State Key Laboratory of Agrobiotechnology, College of Biological Sciences, China Agricultural University, Beijing, 100193 China; 6 King Abdullah University of Science and Technology (KAUST), Thuwal, 23955-6900 Kingdom of Saudi Arabia; 7 Department of Medicine, University of California San Diego, La Jolla, CA 92093 USA

**Keywords:** Dilated cardiomyopathy, NEXN, AAV, Gene therapy, Cardiac function, Disease treatment

## Abstract

**Background:**

Dilated cardiomyopathy (DCM) is one of the most common causes of heart failure. Multiple identified mutations in nexilin (NEXN) have been suggested to be linked with severe DCM. However, the exact association between multiple mutations of Nexn and DCM remains unclear. Moreover, it is critical for the development of precise and effective therapeutics in treatments of DCM.

**Results:**

In our study, Nexn global knockout mice and mice carrying human equivalent G645del mutation are studied using functional gene rescue assays. AAV-mediated gene delivery is conducted through systemic intravenous injections at the neonatal stage. Heart tissues are analyzed by immunoblots, and functions are assessed by echocardiography. Here, we identify functional components of Nexilin and demonstrate that exogenous introduction could rescue the cardiac function and extend the lifespan of Nexn knockout mouse models. Similar therapeutic effects are also obtained in G645del mice, providing a promising intervention for future clinical therapeutics.

**Conclusions:**

In summary, we demonstrated that a single injection of AAV-Nexn was capable to restore the functions of cardiomyocytes and extended the lifespan of Nexn knockout and G645del mice. Our study represented a long-term gene replacement therapy for DCM that potentially covers all forms of loss-of-function mutations in NEXN.

**Supplementary Information:**

The online version contains supplementary material available at 10.1186/s13059-024-03283-x.

## Background

DCM is a progressive cardiac disorder characterized with enlarged ventricular chambers, which leads to heart failure with high incidence and mortality rates worldwide [1, 2]. To date, several studies have discovered different pathogenic DCM-causing mutations in a diverse set of myocardial genes that are passed in either dominantly or recessively to the next generation [3–8]. Current medication of cardiomyopathy mainly relies on chemical drugs, but they can only relieve symptoms, which come with the problem of side effects [9, 10] and financial burden for patients. For advanced disease, pharmacological and device therapy is insufficient to maintain adequate cardiac function, as long-term consecutive treatment either heart transplantation or implantation of long-term mechanical circulatory support is required [11, 12]. Therefore, it is necessary to understand the exact pathogenesis in order to develop the causative therapeutic strategies.

*Nexilin* is a component of the junctional membrane complex that required for the development and maintenance of cardiac T-tubules, and multiple mutations of NEXN has been identified to associate with cardiomyopathy [13–18]. These reports provide the rationale that NEXN is a promising target for clinical care. To date, very little is known about the mechanism behind this association, and no targeted therapy has been developed. Our recent studies showed that global knock out (gKO) of *Nexilin* in mice leads to a rapidly progressive severe DCM, demonstrating that the NEXN is required for the maintenance of normal cardiac structure and function [19]. To further investigate the role of Nexilin in cardiomyopathy, we established a mouse model carrying the glycine deletion at position 645 (G645del, a mutation corresponding to G650del in patients) of the *Nexilin* gene by using the CRISPR/Cas9 system [20]. Accordingly, homozygous G645del mice resulted in a progressive DCM and recapitulated the clinicopathological features that have been observed in patients with the corresponding G650del mutation. These mice with *Nexilin* gene mutations not only are a proper generic model for the study of pathologic progressions but also can be used as an ideal model to discover the novel and effective strategies for the therapeutic interventions of cardiac disease [15, 19, 20].

Adeno-associated virus (AAV)-based vectors are promising vehicles for therapeutic gene delivery [21]. Due to the lack of pathogenicity, low immunogenicity, and durable expression of the therapeutic components even in non-dividing cells such as cardiomyocytes, leading AAV-mediated gene replacement therapy is a promising method in treating cardiac disorders [22–25]. Currently, several AAV serotypes have been identified and applied in both scientific research and clinical treatment [26–30]. Among them, AAV serotype 9 (AAV9) is considered to be the most effective mammalian cardiomyocyte transducer from systemic injections [31, 32]. Based on these, this study investigates the feasibility and effect of a gene therapy method mediated by systemic administration of AAV-encoded expression of Nexilin*.* The present study contributes to a deeper understanding of the relationship between the functional components of Nexn and the pathogenesis of DCM. In addition, it provides insight into the development of better therapeutic methods to restore the normal cardiac function in a causative manner.

## Results

### AAV-mediated *Nexilin* gene replacement rescues cardiac dysfunction and extends lifespan of *Nexn* gKO mice

**Fig. 1:**
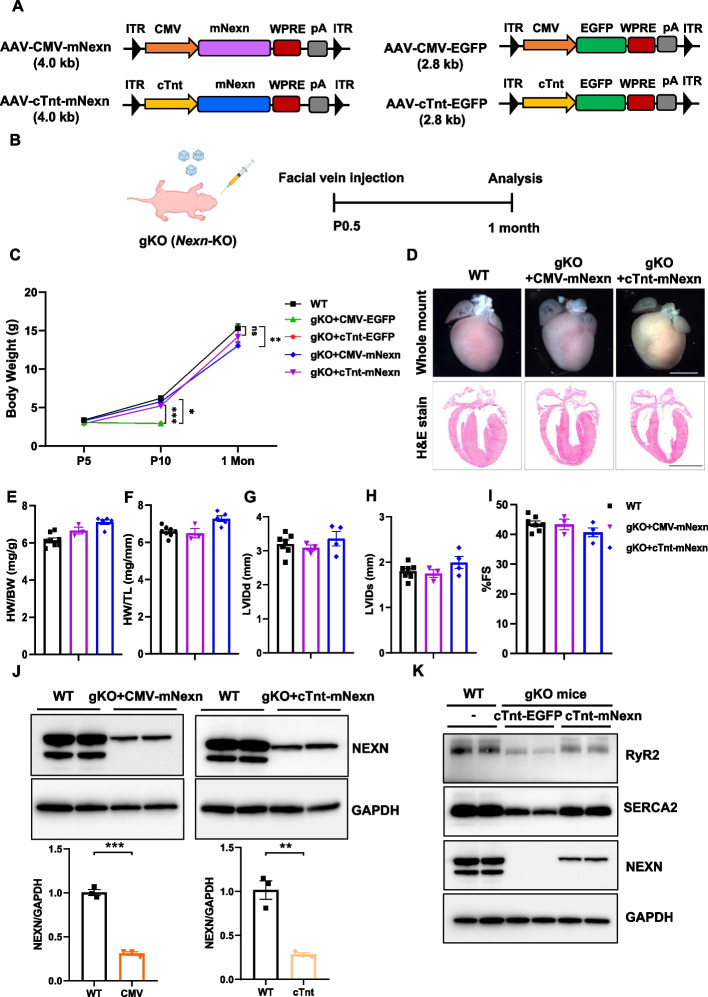
AAV-mediated *Nexilin* gene replacement rescues cardiac dysfunction and extends lifespan of *Nexn* gKO mice. **A** Schematic diagram of AAV constructs. CMV or cTnt promoter drives expression of full-length of murine *Nexilin*. AAVs carrying EGFP driven by either CMV or cTnt were used as controls. **B** The in vivo gene therapy scheme. AAV was injected into postnatal day 0.5 (P0.5) by facial vein, and the following data were collected at 1 month after treatments. **C** Injection of murine Nexilin rescues body weight loss (WT: *n* = 8, CMV-EGFP: *n* = 3, cTnt-EGFP: *n* = 3, CMV-mNexn: *n* = 3 and cTnt-mNexn: *n* = 5). Data are expressed as mean ± SEM. *P < 0.05, **P < 0.01, and ***P < 0.001, Student’s *t*-test. **D** Representative whole-heart H&E-stained images from indicated mouse strains at 1 month after treatment, scale bar: 1 mm. **E**–**I** Transthoracic echocardiographic measurements from WT and gKO mice at 1 month after treatment. **E** Heart mass-to-body weight ratio (HW/BW) (WT: *n* = 8, CMV-mNexn: *n* = 3 and cTnt-mNexn: *n* = 5). **F** Heart weight to tibial length ratio (HW/TL) (WT: *n* = 8, CMV-mNexn: *n* = 3 and cTnt-mNexn: *n* = 5). **G** Left ventricular internal diameter end-diastole (LVIDd) (WT: *n* = 7, CMV-mNexn: *n* = 3 and cTnt-mNexn: *n* = 4). **H** Left ventricular internal diameter end-systole (LVIDs) (WT: *n* = 7, CMV-mNexn: *n* = 3 and cTnt-mNexn: *n* = 4). **I** Percentage of fraction shortening (FS) (WT: *n* = 7, CMV-mNexn: *n* = 3 and cTnt-mNexn: *n* = 4). **J** Protein expression of *N*exilin in cardiac tissue of indicated mouse strains at P5 (postnatal day 5). All proteins were normalized to GAPDH (*n* = 3 each). Only the long isoform of NEXN from the WT was utilized in our AAV construct and quantitated in the bar graphs. Data are expressed as mean ± SEM. **P < 0.01 and ***P < 0.001, Student’s *t*-test. **K** Western blot images of NEXN and Ca^2+^-handling proteins (RyR2 and SERCA2) from WT (postnatal day 10, P10) and gKO mice treated with cTnt promoter-driven EGFP (P10) or murine *N*exilin (P10). gKO, *Nexn* global knockout mice; cTnt, cardiac troponin T promoter

NEXN is an F-actin binding protein and expressed mainly in the heart and skeletal muscle [13]. Complete absence of NEXN leads to premature death in gKO mice within a very narrow time frame [19] (all mice died around 10 days after birth) (Additional file 1: Fig. S1), which represents the childhood onset and early death phenotype in certain human patients with *NEXN* mutations [16]. Although gKO mice exhibited severe cardiomyopathy, it is unclear whether the high mortality of gKO mice is due only to the absence of Nexilin in cardiomyocytes or whether loss of function of Nexilin in other tissues also contributes to this phenotype. To investigate the causality of NEXN in pathology, we produced AAV9 that can ubiquitously express the full-length murine *Nexn* (mNexn) under the control of either CMV promoter (AAV-CMV-mNexn) or cardiac troponin T (cTnt) promoter (AAV-cTnt-mNexn) (Fig. 1A). Due to the early disease onset in *Nexn* gKO mice, we conducted gene delivery in gKO mice at the early neonatal stage (postnatal day 0.5, P0.5). In parallel, AAV-CMV-EGFP and AAV-cTnt-EGFP viruses were produced and independently delivered into P0.5 mice (wild type) through facial vein injections to validate the cardiac specificity of the CMV and cTnT promoters. We then examined the gene expression in different organs 7 days after injections. The results clearly showed that the expression of EGFP driven by cTnT promoter was preferentially restricted to the heart compared with the CMV promoter after systemic administration (Additional file 1: Fig. S2).

To assess the therapeutic effect of AAVs, 1 × 10 [11] genome vectors of AAV-CMV-mNexn or AAV-cTnt-mNexn were delivered into neonatal (P0.5) mice via facial vein injection (Fig. 1B). As expected, the body weight of the control groups of gKO mice administrated either AAV-CMV-EGFP or AAV-cTnt-EGFP virus was significantly reduced, and none of the mice could survive more than 10 days after birth. In contrast, mice received AAV-mNexn steadily gained weight and remained alive for 1 month (Fig. 1C). Interestingly, the mice treated with AAV-CMV-mNexn or AAV-cTnt-mNexn not only could survive over the first 10 days but also showed a similar trend of body weight increase and normal cardiac morphology as wild-type (WT) mice (Fig. 1C, D). In addition, cardiac functions of treated mice were assessed after AAV treatment at the 1-month time point (Fig. 1E–I). Instead of increased ratios of heart weight to body weight and heart weight to tibial length in gKO mice during early development [19], the DCM parameters of Nexn-deficient mice were all at normal levels as in WT mice at 1 month after the AAV-mNexn treatment (Fig. 1E, F). Moreover, transthoracic echocardiography proved that the left ventricular dimensions of AAV-mNexn treated mice were similar to those of the WT group (Fig. 1G, H). Most importantly, the treated mice had a normal percentage of fractional shortening, indicating cardiac function was largely restored after AAV-mNexn treatment (Fig. 1I). To evaluate the transduction efficiency of AAV-Nexn at the protein level, mouse heart tissues were collected for Western blot analysis. Both AAV-CMV-mNexn and AAV-cTnt-mNexn-treated mice restored ~ 30% of Nexilin protein expression as that in WT mice (Fig. 1J). Although the level was not high, it was sufficient for the cardiac function to fully recover after the AAV-mNexn treatments. Moreover, previously we demonstrated that the heart dysfunction in Nexn KO mice was linked to a consequence of Ca^2+^-handling proteins disruption [19]. Here, we observed upregulated expressions of Ca^2+^-handling proteins, including RyR2 and SERCA2, in gKO mice after cardiac-specific induction of murine NEXN (Fig. 1K). Taken together, these data suggest that a partial restoration of Nexilin expression is sufficient to mitigate cardiomyopathy and indicate that the premature or perinatal lethality of gKO mice is mainly attributed to the absence of Nexilin in cardiomyocytes rather than in other tissues.

### Identify Nexilin functional components essential for cardiac functions

**Fig. 2:**
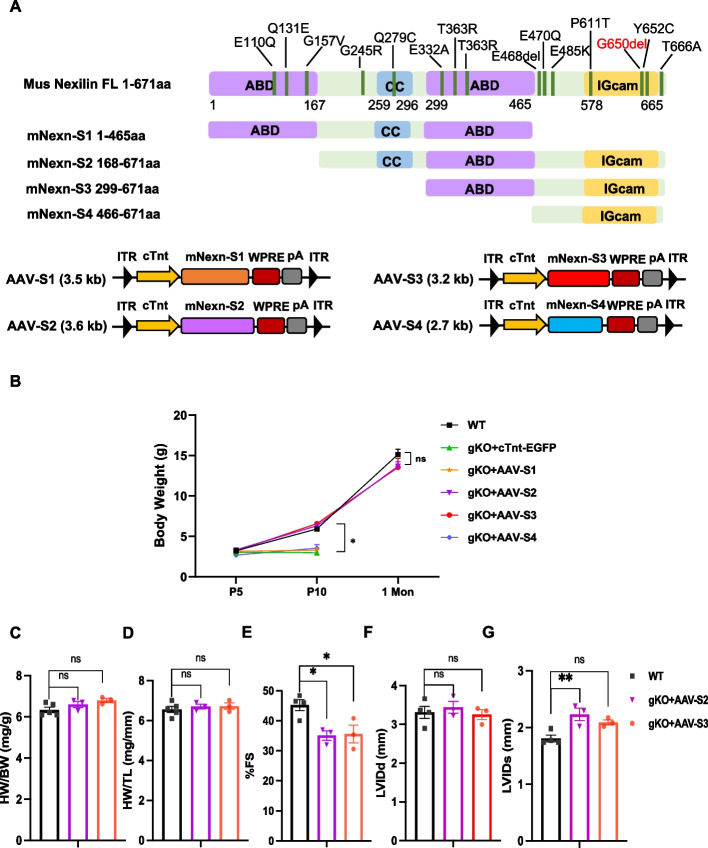
Identify Nexilin functional components responsible for normal cardiac functions. **A** Schematic representation of Nexilin expression constructs. (*Upper*) Full length of Nexilin with 671 amino acids (aa) consists of a central coiled-coil (CC) domain flanked by two actin-binding domains (ABD) and a highly basic C-terminal IGcam domain. S1 (1-465aa) is a shortened version that removes C-terminal Igcam-containing region. S2 (168-671aa) deletes the first ABD domain. S3 (299-671aa) consists of the second ABD domain and IGcam domain. S4 (466-671aa) only contains C-terminal domain with IGcam. Key mutations within the coding region of *NEXN* gene identified from DCM patients are marked. (*Lower*) Schematic diagram of AAV construct containing cTnt promoter-driven different segments of mNexn variants. **B** Body weight changes of wild type and gKO mice injected with empty AAV vector or S1 to S4 variants over time (WT: *n* = 5, cTnt-EGFP: *n* = 5, AAV-S1 to AAV-S4: *n* = 3 each). **C**–**G** Transthoracic echocardiographic measurements of WT and gKO mice 1 month after treatments: **C** HW/BW (WT: *n* = 5, AAV-S2: *n* = 3 and AAV-S3: *n* = 3), **D** HW/TL (WT: *n* = 5, AAV-S2: *n* = 3 and AAV-S3: *n* = 3), **E** FS (WT: *n* = 4, AAV-S2: *n* = 3 and AAV-S3: *n* = 3), **F** LVIDd (WT: *n* = 4, AAV-S2: *n* = 3 and AAV-S3: *n* = 3), and **G** LVIDs (WT: *n* = 4, AAV-S2: *n* = 3 and AAV-S3: *n* = 3). Data are expressed as mean ± SEM. *P < 0.05, **P < 0.01, one-way ANOVA with Tukey’s post hoc multiple comparisons test was performed

Nexilin is a highly conserved protein with 88% identity between mouse and human. It contains two N-terminal actin-binding domains (ABD), a coiled-coil domain, and a C-terminal immunoglobulin superfamily class (IGcam). Multiple mutations in all these domains have been found to associate with cardiomyopathy. (Fig. 2A and Additional file 1: Fig. S3). Our recent study has shown that the first N-terminal ABD domain is not essential for cardiac function, suggesting actin cross-linking activity of NEXN is dispensable for cardiac function [20, 33]. However, it is still unclear which domain in Nexn is essential for its biological function in heart. To explore the physiological function of individual domains of the Nexilin protein, we engineered four different truncated variants and packaged them into AAV9 vector driven by cTnt promoter as AAV-cTnt-mNexn-S1, S2, S3, and S4, respectively (Fig. 2A). 1 × 10 [11] genome copies of each AAV were conducted into different neonatal mouse through facial vein injection. The S1 variant with a deletion of the C-terminal IgCam-containing domain failed to rescue the Nexn gKO mice, and they all died around 10 days after injection (Fig. 2B). In contrast, the gKO mice injected with either the S2 variant (a deletion of the first N-terminal ABD domain) or the S3 variant (deletion of a following coiled-coil domain in S2 variant), but not the mice injected with the S4 variant (deletion of second ABD domain in S3 variant), were able to survive with a comparable body weight to the WT controls. We then examined the heart/body weight ratio and cardiac functions of mice in the S2 and S3 variants treatment groups at 1 month of age (Fig. 2C through G). The results show that C-terminal ABD and IgCam domains of Nexilin are indispensable for sustaining cardiac development and normal cardiac function, whereas the N-terminal ABD and the coiled-coil domains are not. Taken together, our results demonstrated that a short version of Nexilin (S3, containing only an ABD and IGcam domain) could maintain similar functions as the full-length protein, providing an alternate for the Nexn gene replacement therapy. In addition, together with our previously findings and the results showed up here, it also indicated that exon 3–4 skipping strategy could be a therapeutic alternative for patients with their mutations located in the first actin binding domain [20].

### Cardiac-specific induction of human NEXN prevents cardiomyopathy in *Nexn* G645del mice

**Fig. 3:**
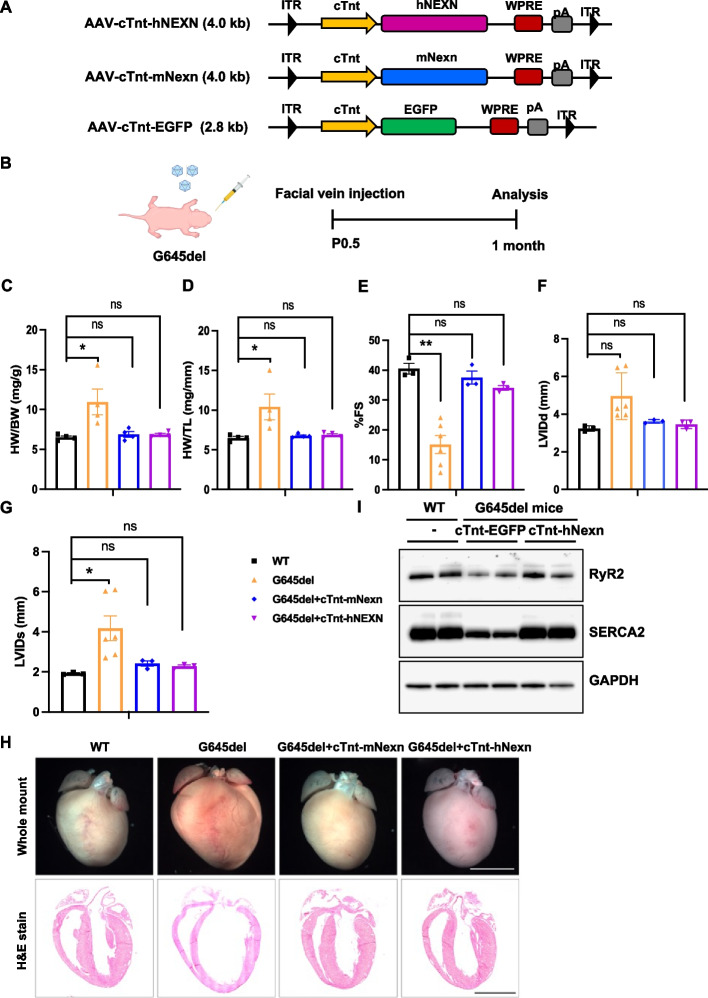
Cardiac-specific induction of human NEXN prevents cardiomyopathy in *Nexn* G645del mice. **A** Schematic view of AAV vector design. Both h*N*EXN and m*N*exn expressions were driven through cTnt promoter and AAV carrying EGFP was used as a control. **B** The in vivo gene therapy scheme. AAV was injected into facial vein of postnatal day 0.5 (P0.5) mouse, and the following data were collected at 1 month after treatments. **C**–**G** Transthoracic echocardiographic measurements in indicated mice 1 month after AAV treatment: **C** HW/BW (*n* = 4 each), **D** HW/TL (*n* = 4 each), **E** FS (WT: *n* = 3, G645del: *n* = 6, cTnt-mNexn: *n* = 3 and cTnt-hNEXN: *n* = 3), **F** LVIDd (WT: *n* = 3, G645del: *n* = 6, cTnt-mNexn: *n* = 3 and cTnt-hNEXN: *n* = 3), and **G** LVIDs (WT: *n* = 3, G645del: *n* = 6, cTnt-mNexn: *n* = 3 and cTnt-hNEXN: *n* = 3). Data are expressed as mean ± SEM. *P < 0.05, **P < 0.01, one-way ANOVA with Tukey’s post hoc multiple comparisons test was performed. **H** Representative whole-heart H&E-stained images from indicated mouse at 1 month after treatment, scale bar: 1 mm. **I** Western blot images of Ca^2+^-handling proteins from WT mouse and G645del mice with or without treatments of human *N*EXN expression driven by cTnt promoter

To further evaluate the therapeutic effect for clinical translation, the G645del mice carrying the corresponding mutation in human patients were applied in our gene replacement experiments by delivering a human copy of *NEXN* through AAV. The G645del mutation is located in the IGcam domain and is conserved among species (Additional file 1: Fig. S3 and S4). The G645del mice exhibit similar pathological features of DCM as the Nexn gKO mice [20]. In this experiment, we generated AAV9 with the full length of human *NEXN* and cTnt promoter (AAV-cTnt-hNEXN; Fig. 3A). In parallel, the AAV with murine Nexn driven by the cTnt promoter was used as a control (AAV-cTnt-mNexn; Fig. 3A). As done in gKO mice, 1 × 10 [11] vector genomes of AAVs were injected into G645del mice through the facial vein at postnatal day 0.5 (Fig. 3B). The control group received an injection of 1 × 10 [11] vector genomes of AAV-cTnt-EGFP (Fig. 3A). One month after AAV transduction, cardiac function of mice from these groups were examined by echocardiogram. Mice treated with AAV-cTnt-EGFP displayed a progressive deterioration of heart function, whereas the cardiac function of G645del mice in the both groups treated with AAV-cTnt-hNEXN and AAV-cTnt-mNexn improved evidently and even recovered to the normal level of healthy WT mice (Fig. 3C–G). In addition, mice treated with AAV-cTnt-mNexn or AAV-cTnt-hNEXN displayed a similar cardiac morphology as WT mice (Fig. 3H). Moreover, the expression of Ca^2+^-handling proteins such as RyR2 and SERCA2 was upregulated in G645del mice treated with cardiomyocyte specific expression of human NEXN (Fig. 3I). Overall, these results indicate that exogenous expression of human NEXN by AAV-mediated gene delivery could prevent cardiomyopathy and restore cardiac function in G645del mice, demonstrating the high feasibility and efficacy of AAV-mediated in vivo gene therapy for DCM that caused by *NEXN* mutations in patients.

### Long-term therapeutic effect of AAV-mediated *Nexilin* gene replacement treatment to *Nexn* gKO mice

**Fig. 4:**
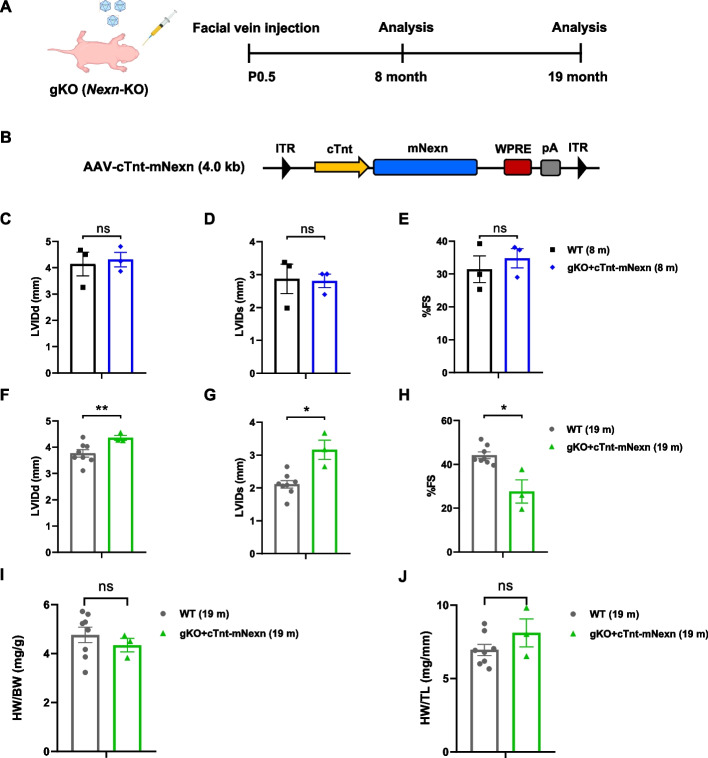
Evaluation of long-term therapeutic effects of AAV-mediated *Nexilin* gene replacement in vivo. **A** Experimental scheme for in vivo experiments. AAV was conducted into postnatal day 0.5 (P0.5) by facial vein injections, and the following data were acquired at 8 months (*n* = 3 each) and 19 months (WT: *n* = 8 and cTnt-nMexn: *n* = 3) after treatments. **B** Schematic diagram of AAV construct; murine *Nexn* expression was driven by cTnt promoter. **C**–**H** Transthoracic echocardiographic measurements in indicated mice after AAV treatment at 8 (**C**–**E**) and 19 months (**F**–**J**): **C**, **F** LVIDd, **D**, **G** LVIDs, **E**, **H** FS. **I**–**J** The heart weight ratio after 19 months of AAV treatments: **I** HW/BW, **J** HW/TL. Data are expressed as mean ± SEM. *P < 0.05, **P < 0.01, and ***P < 0.001, Student’s *t*-test

We also evaluated the long-term therapeutic effect of *Nexilin* gene replacement in vivo. To this end, the neonatal gKO mice were injected via the facial vein with 1 × 10 [11] genome vectors of AAV (Fig. 4A), containing murine *Nexn* driven by the cTnt promoter (AAV-cTnt-mNexn; Fig. 4B). Meanwhile, WT mice were used as controls. Cardiac functions were assessed at the 8th month after treatments with echocardiograms.

Cardiac functions of AAV-cTnt-mNexn-treated mice were restored to normal levels as WT mice at the 8th month after gene delivery (Fig. 4C–E). We also examined the further prolonged therapeutic effects of AAV-cTnt-mNexn-treated mice. Remarkably, these *Nexn* gKO mutant mice, which typically die within 10 days after birth, extended their lifespan by more than one and a half years after a single AAV treatment at P0.5. Although some echocardiographic parameters attenuated somewhat at 19 months (Fig. 4F–H), there were no significant changes between WT and AAV-treated mice in the ratios of heart weight to either body weight (HW/BW) or to tibial length (HW/TL) (Fig. 4I, J). Collectively, these data demonstrated that a single injection of AAV-mNexn was sufficient to improve the cardiac function and extended the lifespan of *Nexn* knockout mice over a long period of time. The results presented here are promising and demonstrated as a potential therapeutic method for the treatment of DCM in the clinic.

## Discussion

Overall, our data showed that AAV9-mediated *Nexilin* gene replacement in cardiomyocytes could effectively alleviate cardiac dysfunction and extend the lifespan of Nexn-deficient mice. In this study, we have demonstrated a promising therapeutic strategy for the treatment of genetic cardiac disease, especially for the cases of morbidity and mortality at a very early stage, allowing only a narrow time window frame for treatments. Particularly, certain NEXN mutations could cause severe fetal cardiomyopathy or early childhood onset in human patients who died within few weeks or months after births [16, 34]. For neonatal patients, early gene therapy through intravenous injection or infusion is the optimal treatment window. Furthermore, recent research in the field of prenatal gene therapy, facilitated by early diagnosis and in-utero gene delivery, illuminates the feasibility of treating severe genetic disorders that manifest early in life [35, 36]. Our strategy has the potential to be applied to homozygous human patients through systemic administration, coupled with cardiomyocyte-specific expression and early interventions.

Notably, more than 15 mutations have been identified in the coding region of NEXN, making the development of a universal and effective gene-editing tool to correct all the mutations quite challenging. By comparison, the gene replacement therapy strategy that we developed showed promising and high safety for its relevant DCM therapeutics. In addition, our results showed that the truncated variant S3, which contains only a single ABD domain and a C-terminal IGcam domain, was sufficient to sustain the normal function of the Nexilin protein, which may provide an alternative option of *N*exilin gene therapy for the treatment of DCM. This opened a new insight into cases where multiple genes need to be co-delivered simultaneously, which could overload the limitation of a single AAV packaging capacity (~ 4.7 kb).

Interestingly, we observed that a small portion (~ 30%) of Nexn protein expression could extend lifespan and almost completely restored the cardiac functions in *Nexn*-defective DCM mice. This result is consistent with our previous findings that, compared with WT controls, heterozygous gKO mice and a mouse line with deletion of *Nexn* exon 3 and 4 (e3-4del) had normal heart function despite expressing only 50% and 20% amounts of Nexn protein, respectively [20]. Our results showed that the restored heart function persisted for 8 months after AAV treatments, although the functional index declined somehow after 19 months. This might indicate the general fate of AAV treatments, because recombinant AAV is diluted over time due to the property of non-integration, leading to a gradual loss of the transgene and its expression [37, 38]. Nevertheless, our data showed that the lifespan and the normal heart/body weight ratio of treated mice could be maintained for up to 19 months after this single dose of AAV treatment. Furthermore, the efficacy of human NEXN gene replacement therapy in treating G645del mice, which have similar clinical pathological and genetic features to human DCM patients, provided a promising intervention for further clinical therapeutics. In summary, our study represents a long-term gene replacement therapy for DCM that can potentially cover all forms of Nexilin loss-of-function mutations.

## Conclusions

Systemic delivery of AAV9-Nexn or even its C-terminal short version could effectively restore cardiac functions in both a global knock out and a clinically relevant single glycine deletion DCM animal model. Furthermore, a single AAV treatment was sufficient to extend the lifespan of Nexn mutants from untreated neonatal deaths to more than 19 months.

## Materials and methods

### AAV vectors and cloning

We generated nine AAV vectors in total, which were named AAV-CMV-mNexn, AAV-CMV-EGFP, AAV-cTnt-mNexn, AAV-cTnt-EGFP, AAV-cTnt-mNexn-S1, AAV-cTnt-mNexn-S2, AAV-cTnt-mNexn-S3, AAV-cTnt-mNexn-S4, and AAV-cTnt-hNEXN, respectively. To amplify the sequences encoding human and mouse Nexilin, total RNAs were extracted from human and mouse cardiomyocytes, respectively, and converted into cDNA for their cloning. AAV vectors were prepared by using endotoxin-free plasmid DNA purification kit (Takara, Cat. 740,548).

### AAV production

AAV2/9 (AAV2 inverted terminal repeat (ITR) vectors pseudo-typed with AAV9 capsid) viral particles were generated by or following the procedures of the Gene Transfer Targeting and Therapeutics Core at the Salk Institute for Biological Studies. AAV titration was done by using qPCR (ITR-F primer: GGAACCCCTAGTGATGGAGTT, ITR-R primer: CGGCCTCAGTGAGCGA). AAV genome was extracted through alkaline buffer (25 nM NaOH, 0.2 mM EDTA) digestion at 98 °C for 8 min; reaction was terminated by neutralization buffer (40 mM Tris–Hcl, pH 5.0, 0.005% Tween 20). Then, we performed the serial dilution of standard and sample AAV for qPCR.

### Animals

All gKO mice and G645del mice were generated as previously described [19, 20]. When homozygous gKO and G645del mice were obtained at the first day after birth, they were subjected to the experiment. All animals were housed under a 12-h light cycle with ad libitum access to food and water and maintained by UCSD Animal Care Program. All experimental procedures were approved by the IACUC committee of UCSD. Primers (5′-3′) for genotyping are listed as below.

For gKO mice:

- *Nexilin* forward primer: TCAAAGGGAAGGTCATTAAAATTC
- *Nexilin* reverse primer (For WT allele): TGATGATGATGATGTTGCTAAGTG
- *Nexilin* reverse primer (For KO allele): CAAAGATACCAAGAAAAGTTGGGA

For G645del mice:

- G645 WT forward primer: CTTCCCAGAAGATGGAGGA
- G645del forward primer: CTTCCCAGAAGATGGAGAG
- G645 reverse primer (common use): AGCATGGTAATGAACCTGATATGC

### Facial vein AAV injection

Facial vein injection was conducted into newborn mice (P0-P1) as described [39]. 1 × 10 [11] genome copies of AAV were used in the volume of 30 µL for injection.

### Echocardiography

For echocardiography, mice were anesthetized with 3% isoflurane for 10 s and maintained at a 0.5% isoflurane during the procedure. Echocardiography was performed by using a VisualSonics, UJIFILM Sonosite, Vevo 2100 ultrasound system with a linear transducer 32–55 MHz.

### Whole heart imaging and histology

Heart tissues were collected and perfused with PBS and then fixed overnight in 4% PFA. Afterwards, they were proceeded for heart imaging and histology as described before [7].

### Western blot

Total protein extracts were lysed in RIPA buffer (50 mM Tris, 150 mM sodium chloride, 2% Nonidet P-40, 0.1% SDS, 0.25% deoxycholic acid, 10 mM EDTA, 0.01% Sodium azide). Protein lysates were separated on 4–12% SDS-PAGE gels (Life Technologies) and transferred to PVDF membrane (BioRad). After 5% dry milk blocking for 1 h, membranes were incubated with indicated primary antibodies overnight at 4 °C. Blots were washed with TBST and incubated with HRP-conjugated secondary antibodies for 1–2 h at room temperature. Immunoreactive bands were visualized using an enhanced chemiluminescence reagent (Thermo) following manufacturer instructions. At least two independent experiments were performed for each antibody. List of antibodies is as follows: RyR2 (ENZO, ALX-804–016-R100), SERCA2 (Santa Cruz, sc-73022), GAPDH (Santa Cruz, sc-32233).

### Real-time PCR

Tissues from mice were homogenized in liquid nitrogen, and the total RNA was extracted with TRIzol reagent (Invitrogen). qPCR was performed with SYBR Green.

The primers for qPCR (5′-3′):

- hNEXN forward primer: ATGAATGATATTTCCCAAAAGG
- hNEXN reverse primer: TTAATTCTTACTTTCAATGGTAA
- mNexn forward primer: ATGAATGACGTTTCGCAAAA
- mNexn reverse primer: CTAGTAGTCATCCATTTCAATG
- actin forward primer: GTACGCCAACACAGTGCTG
- actin reverse primer: CGTCATACTCCTGCTTGCTG
- EGFP forward primer: CACAAGTTCAGCGTGTCCG
- EGFP reverse primer: CTCGATGCGGTTCACCAG

### Supplementary Information

Additional file 1: Fig. S1. Schematic illustration of the Cre/LoxP-mediated *Nexn* knockout mice. Fig. S2. Comparison of CMV and cTnt promoters driven gene expressions in different mouse tissues. Fig. S3. Comparison of human and mouse Nexilin protein sequences. Fig. S4. Nexilin G645del mouse.

Additional file 2: Uncropped images of Western blots in Fig. 1 and Fig. 3.

Additional file 3: Raw data for figures.

Additional file 4: Review history.

## Data Availability

The data generated in this study are available within the article and its additional files.
