# Supplementary material for: In vivo rescue of genetic dilated cardiomyopathy by systemic delivery of nexilin
Source: Genome Biol. 2024 May 23;25:135. doi: 10.1186/s13059-024-03283-x (PMC11112773; doi:10.1186/s13059-024-03283-x)
Supplement: Supplementary file 1 — Additional file 1: Fig. S1. Schematic illustration of the Cre/LoxP-mediated Nexn knockout mice. Fig. S2. Comparison of CMV and cTnt promoters driven gene expressions in different mouse tissues. Fig. S3. Comparison of human and mouse Nexilin protein sequences. Fig. S4. Nexilin G645del mouse. [file 13059_2024_3283_MOESM1_ESM.docx]

**Fig. S1. Schematic illustration of the Cre/LoxP-mediated *Nexn* knockout mice.**

Use genome-targeting approach to introduce the insertion of LoxP within *Nexilin* gene. *Nexn*^fl/fl^ mice were crossed to Cre transgenic mice, which expressed Cre under Sox2 promoter. Cre promoted recombination between two LoxP sites for *Nexilin* gene knockout. Red triangle indicates the LoxP sites.

**Fig. S2. Comparison of CMV and cTnt promoters driven gene expressions in different mouse tissues.**

(**A**) Fluorescent images of EGFP under the control of cTNT promoter segment or constitutive CMV promoter in various tissues. The organs were harvested from 7 dpp (days postpartum) mice injected at P0.5. The numbers at the upper left corner denote the exposure times of fluorescent images. Blank: no injection. (**B**) qRT-PCR analysis of EGFP induction in different tissues of mice that received facial vein injections of AAV-CMV-EGFP or AAV- cTnt-EGFP at 7 dpp. Relative expression level of EGFP was normalized to GAPDH and set as 100 in the tissue with highest expression (i.e., liver for AAV-CMV-EGFP and heart for AAV-cTnt-EGFP), and signals in other tissues were normalized accordingly.

**Fig. S3. Comparison of human and mouse Nexilin protein sequences.**

Amino-acid sequence alignment shows high conservation of Nexilin protein between mouse and human. Actin-binding domain (ABD), coiled-coil and IGcam domain are indicated. Black boxes indicate identical amino acids; red bases indicate different amino acids; the position of G650del mutation found in DCM patients is indicated by green box.

**Fig. S4. Nexilin G645del mouse.**

(**A**) A scheme showing the generation of G645del mutant mouse by the CRISPR/Cas9 genome editing approach. sgRNA targeting the WT *N*exilin sequence is indicated by the red line and the PAM sequence is labeled in blue. 3-bp deletion results in a glycine deletion in position 645. (**B**) Sequencing results of the targeted *N*exilin site of WT and G645del mice. The red box indicates the 3 missing base pairs in the G645del mouse. (**C**) Amino acid alignment of partial sequence of the NEXN IGcam domain in 10 species. The corresponding position of G650del mutation found in DCM patients is indicated by red frame.
