# Supplementary figures and images for: In vivo rescue of genetic dilated cardiomyopathy by systemic delivery of nexilin
Source: Genome Biol. 2024 May 23;25:135. doi: 10.1186/s13059-024-03283-x (PMC11112773; doi:10.1186/s13059-024-03283-x)

## Slide 1
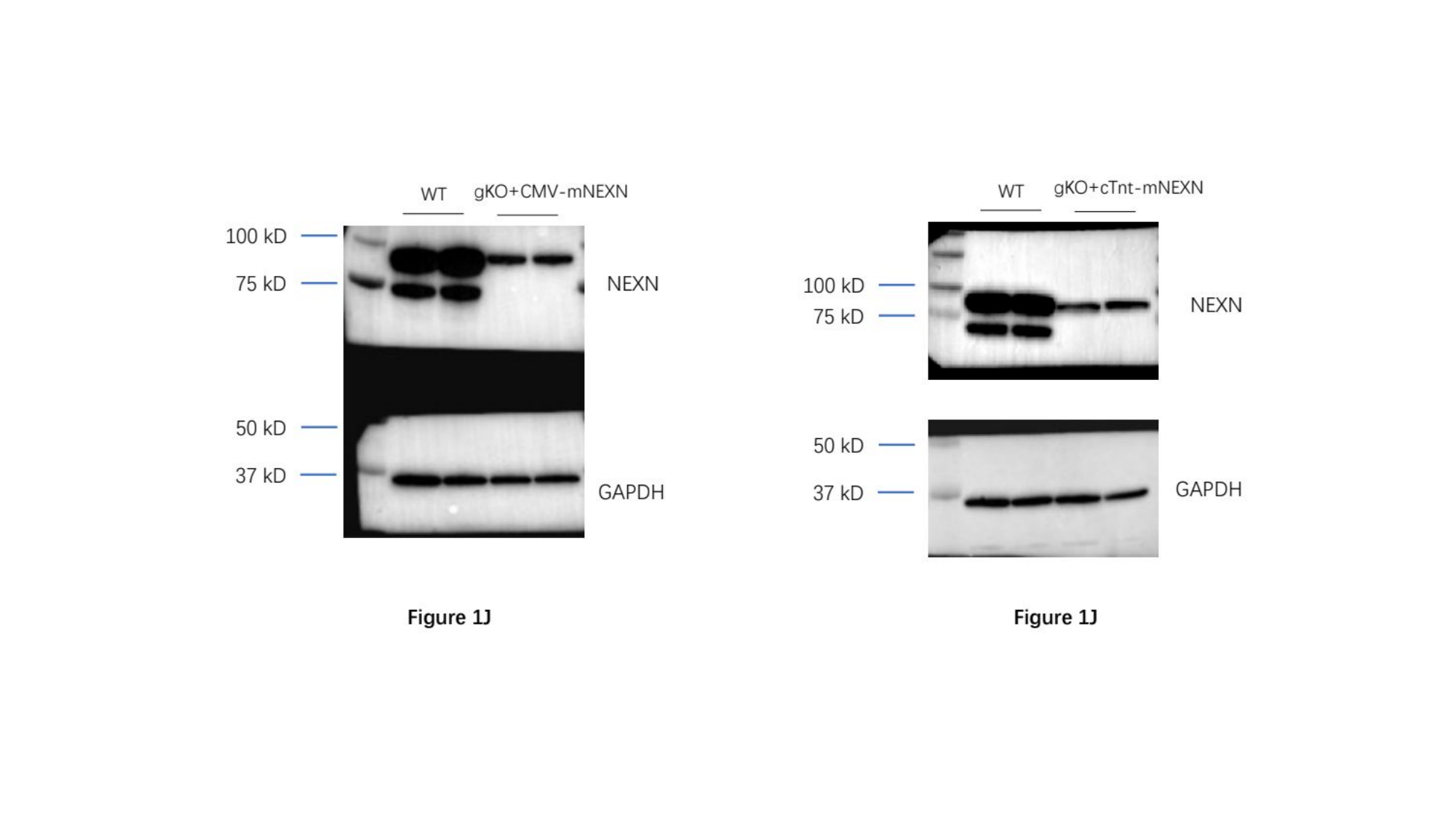

## Slide 2
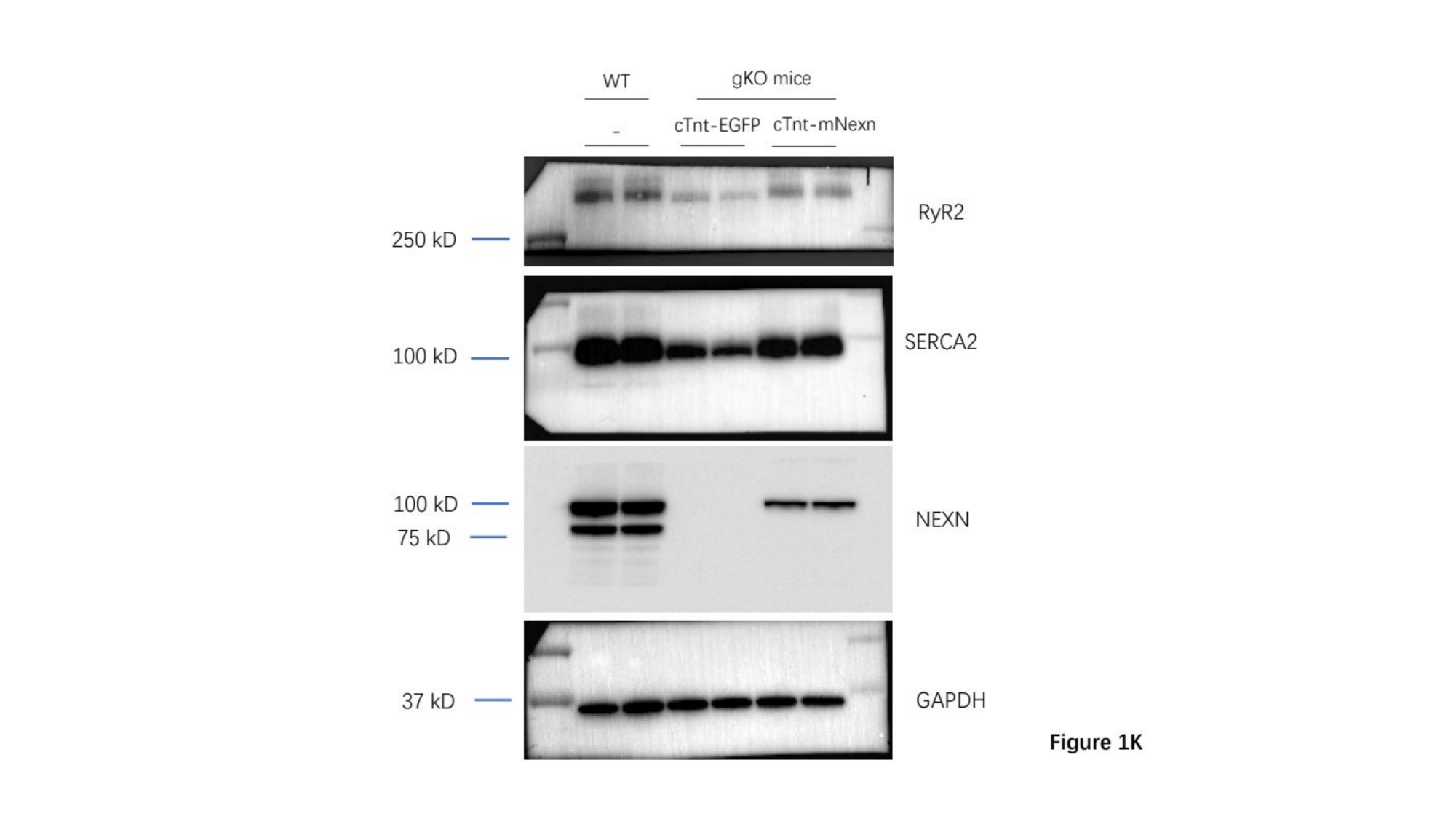

## Slide 3
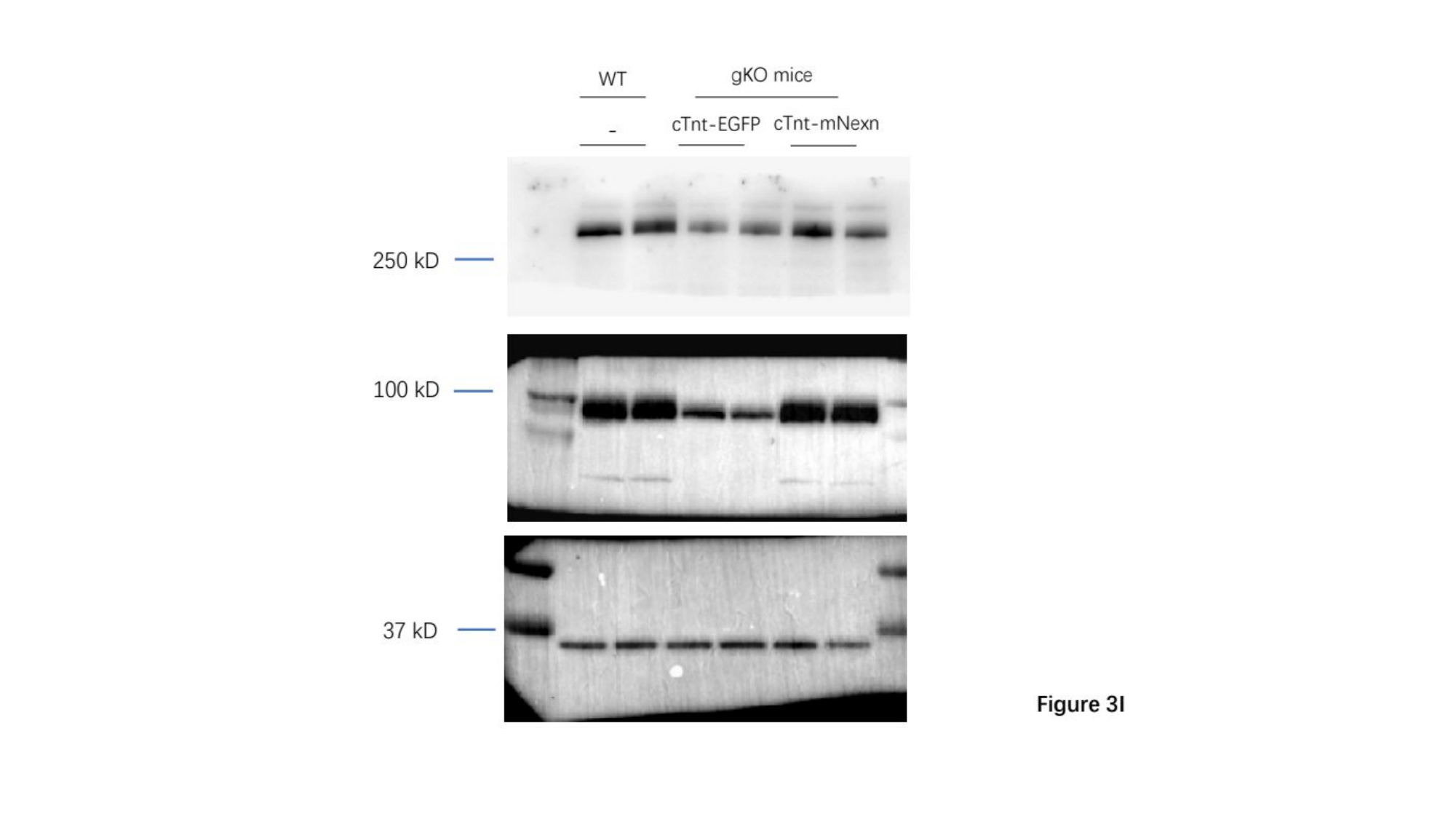

Supplement: Supplementary file 2 — Additional file 2: Uncropped images of Western blots in Fig. 1 and Fig. 3. [file 13059_2024_3283_MOESM2_ESM.pptx]
