# Supplementary material for: In vivo rescue of genetic dilated cardiomyopathy by systemic delivery of nexilin
Source: Genome Biol. 2024 May 23;25:135. doi: 10.1186/s13059-024-03283-x (PMC11112773; doi:10.1186/s13059-024-03283-x)
Supplement: Supplementary file 4 — Additional file 4: Review history. [file 13059_2024_3283_MOESM4_ESM.docx]

Review History

**First round of review**

**Reviewer 1**

**Are you able to assess all statistics in the manuscript, including the appropriateness of statistical tests used?** No, I do not feel adequately qualified to assess the statistics.

**Comments to author:**

In this manuscript, the authors employed AAV-mediated gene delivery in neonatal Nexn global knockout mice and observed that partial restoration of Nexilin expression is adequate to alleviate cardiomyopathy. Furthermore, they delineated the functional components of the Nexn gene, highlighting the indispensability of the C-terminal ABD and IgCam domains of Nexilin for sustaining cardiac development and normal cardiac function, while the N-terminal ABD and coiled-coil domains are not essential. Subsequently, they conducted AAV-mediated gene delivery of human NEXN in Nexn G645del mice, demonstrating that exogenous expression could prevent cardiomyopathy and restore cardiac function in these mice. Lastly, through a single injection of AAV-mNexn into Nexn knockout mice, they observed significant improvement in cardiac function and an extension in lifespan over a prolonged period.

This manuscript furnishes proof-of-concept evidence suggesting that AAV-mediated Nexn gene delivery holds promise as a therapeutic approach for the treatment of DCM resulting from loss-of-function mutations in the NEXN gene. The manuscript is well-crafted, featuring well-designed experiments and robust data. While AAV-mediated gene delivery is not a novel concept, the findings presented here offer valuable insights for the field.

**Reviewer 2**

**Are you able to assess all statistics in the manuscript, including the appropriateness of statistical tests used?** Yes, and I have assessed the statistics in my report.

**Comments to author:**

This manuscript extends upon two previously published studies (PMID: 30982350, 32635769, 32814711), which demonstrate the necessity of NEXN (nexilin) in preventing cardiomyopathy in the early neonatal period. The authors demonstrate that systemic delivery of Nexn or NEXN via the facial vein at P0.5 can rescue the lethal cardiomyopathy observed in Nexn-deficient animals using AAV2/9-mediated gene transfer. Moreover, AAV-Nexn treatment can rescue homozygous Nexn-G645del mice, previously characterized, alleviating their cardiomyopathy. Cardiomyopathy rescue in both mouse models correlated with enhanced expression of calcium-handling genes. Notably, the phenotype rescue following AAV-mediated gene transfer occurred despite only achieving approximately 30% nexilin protein expression compared to control animals, and the clinical benefit persisted for over a year. The rescue is likely cardiomyocyte-autonomous, as viral expression of Nexn using the cardiomyocyte-specific cTnt promoter was as effective as using the ubiquitous CMV promoter. The authors further demonstrated that the mNexn-S3 299-671 amino acid (aa) miniature form of Nexilin, which includes the C-terminal ABD and IgCam domains, is also capable of rescuing gKO mice. Overall, this is a well-executed and compelling study that unveils the therapeutic potential of AAV-NEXN gene therapy for inherited cardiomyopathy, serving as a promising intervention for future clinical therapeutics. I only have a few comments listed below to further improve the manuscript.

1. The work on gKO mice effectively prevents neonatal death. In human patients, homozygous loss of nexilin typically causes early childhood death or lethal fetal cardiomyopathy（PMID: 33949776, 35166435）. Can the authors comment on a vision for AAV gene therapy in homozygous patients?

2. Western blots of wild-type (WT) heart tissue reveal two distinct NEXN isoforms, evidenced by the presence of a second, lower molecular weight band, does nexilin have different expressed isoforms/splicing in mouse heart? Is this second lower band included in the quantitative bar graphs below the blots?

3. Given the rapid mortality of gKO mice by P10, the ability of gene therapy to rescue these mice swiftly and reversibly is remarkable. Considering the temporal progression of AAV infection, encompassing endosomal escape, second-strand synthesis, and protein production and incorporation, have the authors assessed NEXN expression at the earlier timepoints?

4. Please describe the vector production purification methods used, titers obtained, and the volumes used for facial vein injections in the Methods Section.

5. Student's t-test is not an appropriate statistical test for assessing differences between more than two groups (e.g. Figure 2 C-G, Figure 3 C-G); ANOVA should be considered instead.

6. Figure legends should specify the number of animals providing data for each data point.

**Authors Response**

**Point-by-point responses to the reviewers’ comments:**

*We appreciate the reviewers’ recognition of our work and their constructive suggestions. Our revised manuscript now incorporates their feedback, and you can refer to our responses (highlighted in blue) alongside their comments.*

REVIEWER COMMENTS

Reviewer #1: In this manuscript, the authors employed AAV-mediated gene delivery in neonatal Nexn global knockout mice and observed that partial restoration of Nexilin expression is adequate to alleviate cardiomyopathy. Furthermore, they delineated the functional components of the Nexn gene, highlighting the indispensability of the C-terminal ABD and IgCam domains of Nexilin for sustaining cardiac development and normal cardiac function, while the N-terminal ABD and coiled-coil domains are not essential. Subsequently, they conducted AAV-mediated gene delivery of human NEXN in Nexn G645del mice, demonstrating that exogenous expression could prevent cardiomyopathy and restore cardiac function in these mice. Lastly, through a single injection of AAV-mNexn into Nexn knockout mice, they observed significant improvement in cardiac function and an extension in lifespan over a prolonged period.

This manuscript furnishes proof-of-concept evidence suggesting that AAV-mediated Nexn gene delivery holds promise as a therapeutic approach for the treatment of DCM resulting from loss-of-function mutations in the NEXN gene. The manuscript is well-crafted, featuring well-designed experiments and robust data. While AAV-mediated gene delivery is not a novel concept, the findings presented here offer valuable insights for the field.

*Response: We are so grateful to receive the recognition from reviewer #1.*

Reviewer #2: This manuscript extends upon two previously published studies (PMID: 30982350, 32635769, 32814711), which demonstrate the necessity of NEXN (nexilin) in preventing cardiomyopathy in the early neonatal period. The authors demonstrate that systemic delivery of Nexn or NEXN via the facial vein at P0.5 can rescue the lethal cardiomyopathy observed in Nexn-deficient animals using AAV2/9-mediated gene transfer. Moreover, AAV-Nexn treatment can rescue homozygous Nexn-G645del mice, previously characterized, alleviating their cardiomyopathy. Cardiomyopathy rescue in both mouse models correlated with enhanced expression of calcium-handling genes. Notably, the phenotype rescue following AAV-mediated gene transfer occurred despite only achieving approximately 30% nexilin protein expression compared to control animals, and the clinical benefit persisted for over a year. The rescue is likely cardiomyocyte-autonomous, as viral expression of Nexn using the cardiomyocyte-specific cTnt promoter was as effective as using the ubiquitous CMV promoter. The authors further demonstrated that the mNexn-S3 299-671 amino acid (aa) miniature form of Nexilin, which includes the C-terminal ABD and IgCam domains, is also capable of rescuing gKO mice. Overall, this is a well-executed and compelling study that unveils the therapeutic potential of AAV-NEXN gene therapy for inherited cardiomyopathy, serving as a promising intervention for future clinical therapeutics. I only have a few comments listed below to further improve the manuscript.

1. The work on gKO mice effectively prevents neonatal death. In human patients, homozygous loss of nexilin typically causes early childhood death or lethal fetal cardiomyopathy（PMID: 33949776, 35166435）. Can the authors comment on a vision for AAV gene therapy in homozygous patients?

*Response: Thanks for the great comments by the reviewer. For neonatal patients, early gene therapy through intravenous (IV) injection or infusion is the optimal treatment window. Furthermore, recent research in the field of prenatal gene therapy, facilitated by early diagnosis and in-utero gene delivery, illuminates the possibility of treating severe genetic disorders that manifest early in life (PMID: 34618719, 35371822). Our strategy has the potential to be applied to homozygous human patients through systemic administration, coupled with cardiomyocyte-specific expression and early interventions. The findings presented in our study provide strong evidence to support the therapeutic potential of AAV-mediated NEXN gene replacement for patients, particularly for those carrying NEXN loss-of-function mutations. This aspect has been incorporated into the Discussion section of our revised manuscript (Page 9-10).*

2. Western blots of wild-type (WT) heart tissue reveal two distinct NEXN isoforms, evidenced by the presence of a second, lower molecular weight band, does nexilin have different expressed isoforms/splicing in mouse heart? Is this second lower band included in the quantitative bar graphs below the blots?

*Response: We appreciate the reviewer's valuable feedback. Our previous data has demonstrated that NEXN has two major isoforms in cardiomyocytes, a long isoform with 671 amino acids and a short isoform with 657 amino acids (PMID: 30982350). In this study, we used the long isoform for AAV construct and packaging. Therefore, the lower band was not included in the quantitative bar graphs. We have now provided this information in the figure legends.*

3. Given the rapid mortality of gKO mice by P10, the ability of gene therapy to rescue these mice swiftly and reversibly is remarkable. Considering the temporal progression of AAV infection, encompassing endosomal escape, second-strand synthesis, and protein production and incorporation, have the authors assessed NEXN expression at the earlier timepoints?

*Response: We extend our gratitude to the reviewer for this valuable suggestion. Considering the early onset phenotype and the swift mortality of Nexn gKO mice, we did test the AAV-driven gene expression in various mouse tissues at 7 dpp (days postpartum), our data revealed GFP expression was induced with high efficiency in the heart and liver using the CMV promoter, the expression of GFP driven by cTnT promoter was preferentially restricted to the heart compared with the CMV promoter after systemic administration (Supplementary Figure 2). Furthermore, we also specifically assessed the expression of NEXN in heart at P5 (postnatal day 5) stage, the results revealed the expression of NEXN was restored to approximately 30% of WT level under the control of both CMV promoter and cTnT promoter (Figure 1J). We have now explicitly included the timepoint information in the corresponding figure legends.*

4. Please describe the vector production purification methods used, titers obtained, and the volumes used for facial vein injections in the Methods Section.

*Response: We appreciate the reviewer’s comments. We have added the detailed information in the Methods Section.*

5. Student's t-test is not an appropriate statistical test for assessing differences between more than two groups (e.g. Figure 2 C-G, Figure 3 C-G); ANOVA should be considered instead.

*Response: We thank the reviewer for the great comment. We have changed the statistical tests for assessing differences in revised figures and the corresponding figure legends.*

6. Figure legends should specify the number of animals providing data for each data point.

*Response: We appreciate the reviewer’s comment. We have added the animal number into each data point.*
